# Adiposity distribution and risks of 12 obesity-related cancers: a Mendelian randomization analysis

**DOI:** 10.1093/jnci/djaf201

**Published:** 2025-09-24

**Authors:** Emma Hazelwood, Lucy J Goudswaard, Matthew A Lee, Marina Vabistsevits, Dimitri J Pournaras, Hermann Brenner, Daniel D Buchanan, Stephen B Gruber, Andrea Gsur, Li Li, Ludmila Vodickova, Robert C Grant, N Jewel Samadder, Nicholas J Timpson, Marc J Gunter, Benjamin Schuster-Böckler, James Yarmolinsky, Tom G Richardson, Heinz Freisling, Neil Murphy, Emma E Vincent

**Affiliations:** MRC Integrative Epidemiology Unit, University of Bristol, Bristol, United Kingdom; Population Health Sciences, Bristol Medical School, University of Bristol, Bristol, United Kingdom; MRC Integrative Epidemiology Unit, University of Bristol, Bristol, United Kingdom; Population Health Sciences, Bristol Medical School, University of Bristol, Bristol, United Kingdom; Nutrition and Metabolism Branch, International Agency for Research on Cancer (IARC- WHO), Lyon, France; MRC Integrative Epidemiology Unit, University of Bristol, Bristol, United Kingdom; Population Health Sciences, Bristol Medical School, University of Bristol, Bristol, United Kingdom; Clinical and Biomedical Sciences, University of Exeter, Exeter, United Kingdom; Department of Upper GI and Bariatric/Metabolic Surgery, North Bristol NHS Trust, Southmead Hospital, Bristol, United Kingdom; Division of Clinical Epidemiology and Aging Research, German Cancer Research Center (DKFZ), Heidelberg, Germany; Division of Preventive Oncology, German Cancer Research Center (DKFZ) and National Center for Tumor Diseases (NCT), Heidelberg, Germany; German Cancer Consortium (DKTK), German Cancer Research Center (DKFZ), Heidelberg, Germany; Colorectal Oncogenomics Group, Department of Clinical Pathology, Melbourne Medical School, The University of Melbourne, Parkville, Australia; University of Melbourne Centre for Cancer Research, The University of Melbourne, Parkville, Australia; Genomic Medicine and Family Cancer Clinic, The Royal Melbourne Hospital, Parkville, Victoria, Australia; Department of Medical Oncology and Therapeutics Research and Center for Precision Medicine, City of Hope National Medical Center, Duarte, CA, United States; Center for Cancer Research, Medical University of Vienna, Vienna, Austria; Department of Family Medicine, University of Virginia, Charlottesville, VA, United States; Institute of Biology and Medical Genetics, First Faculty of Medicine, Charles University, Prague, Czech Republic; Faculty of Medicine and Biomedical Center in Pilsen, Charles University, Pilsen, Czech Republic; Division of Medical Oncology and Hematology, Princess Margaret Cancer Centre, Toronto, Ontario, Canada; Division of Gastroenterology, Mayo Clinic, Phoenix, AZ, United States; MRC Integrative Epidemiology Unit, University of Bristol, Bristol, United Kingdom; Population Health Sciences, Bristol Medical School, University of Bristol, Bristol, United Kingdom; Nutrition and Metabolism Branch, International Agency for Research on Cancer (IARC- WHO), Lyon, France; Department of Epidemiology and Biostatistics, School of Public Health, Imperial College London, London, United Kingdom; Ludwig Institute for Cancer Research, University of Oxford, Oxford, United Kingdom; Department of Epidemiology and Biostatistics, School of Public Health, Imperial College London, London, United Kingdom; MRC Integrative Epidemiology Unit, University of Bristol, Bristol, United Kingdom; Population Health Sciences, Bristol Medical School, University of Bristol, Bristol, United Kingdom; Nutrition and Metabolism Branch, International Agency for Research on Cancer (IARC- WHO), Lyon, France; Nutrition and Metabolism Branch, International Agency for Research on Cancer (IARC- WHO), Lyon, France; MRC Integrative Epidemiology Unit, University of Bristol, Bristol, United Kingdom; Population Health Sciences, Bristol Medical School, University of Bristol, Bristol, United Kingdom; Translational Health Sciences, Bristol Medical School, University of Bristol, Bristol, United Kingdom

## Abstract

**Introduction:**

There is convincing evidence that overall adiposity increases the risks of several cancers. Whether the distribution of adiposity plays a similar role is unclear.

**Methods:**

We used 2-sample Mendelian randomization (MR) to examine causal relationships of 5 adiposity distribution traits (abdominal subcutaneous adipose tissue (ASAT); visceral adipose tissue (VAT); gluteofemoral adipose tissue (GFAT); liver fat; and pancreas fat) with the risks of 12 obesity-related cancers (endometrial, ovarian, breast, colorectal, pancreas, multiple myeloma, liver, kidney (renal cell), thyroid, gallbladder, esophageal adenocarcinoma, and meningioma).

**Results:**

Sample size across all genome-wide association studies (GWAS) ranged from 8407 to 728 896 (median: 57 249). We found evidence that higher genetically predicted ASAT increased the risks of endometrial cancer, liver cancer, and esophageal adenocarcinoma (odds ratios (OR) and 95% confidence intervals (CI) per standard deviation (SD) higher ASAT = 1.79 (1.18 to 2.71), 3.83 (1.39 to 10.53), and 2.34 (1.15 to 4.78), respectively). Conversely, we found evidence that higher genetically predicted GFAT decreased the risks of breast cancer and meningioma (ORs and 95% CIs per SD higher genetically predicted GFAT = 0.77 (0.62 to 0.97) and 0.53 (0.32 to 0.90), respectively). We also found evidence for an effect of higher genetically predicted VAT and liver fat on increased liver cancer risk (ORs and 95% CIs per SD higher genetically predicted adiposity trait = 4.29 (1.41 to 13.07) and 4.09 (2.29 to 7.28), respectively).

**Discussion:**

Our analyses provide novel insights into the relationship between adiposity distribution and cancer risk. These insights highlight the potential importance of adipose tissue distribution alongside maintaining a healthy weight for cancer prevention.

## Introduction

The prevalence of obesity is increasing worldwide, having doubled in more than 70 countries over the past 4 decades with 4 billion individuals estimated to be living with obesity by 2035.^1^^,^^2^ Obesity is known to increase the risk of cancer, which is the second most common cause of death worldwide.^3^ In 2016, an International Agency for Research on Cancer (IARC) report concluded that there is sufficient evidence to support an association of obesity with the risks of at least 13 cancers—endometrial, ovarian, breast, colorectal, pancreas, multiple myeloma, liver, kidney (renal cell), thyroid, gallbladder, esophageal adenocarcinoma, gastric cardia, and meningioma.^4^ A growing body of evidence shows that effective obesity care can lead to a reduction in cancer risk.^5-12^ However, the importance of the anatomical distribution of adiposity in the obesity-cancer risk relationship is not fully understood.

Adipose tissue, primarily consisting of adipocytes, is distributed throughout the body and plays a crucial role in energy storage and metabolism.^13^^,^^14^ Additionally, it functions as an endocrine organ, secreting hormones and circulatory factors.^15^ Each deposit originates from a distinct fat progenitor and has a unique microenvironment and cytological makeup.^16^^,^^17^ For instance, visceral adipose tissue has an important role in the secretion of inflammation-related adipokines and growth factors, whereas subcutaneous adipose tissue is the predominant source of circulating estrogen in men and postmenopausal women.^18^^,^^19^ Furthermore, each deposit interacts with different organs and biological structures proximal to the adipose tissue.^20^

Body mass index (BMI) is often used as a proximal measure of adiposity in epidemiological studies. As a simple anthropometric derivative (height for stature) correlated with many other body composition traits, BMI is useful in large-scale population studies. However, BMI cannot capture the distribution of adipose tissue throughout the body.^21-23^ Recent research has revealed that more specific adiposity distribution traits, including abdominal subcutaneous, visceral, and gluteofemoral adipose tissue (ASAT, VAT, and GFAT, respectively), as well as the fat content of certain organs, in particular the liver and pancreas, have been shown to have differential roles in the effect of adiposity on risk of cardiometabolic outcomes.^21^^,^^24^^,^^25^ Such findings have led to important updates in clinical guidelines. For example, the European Association for the Study of Obesity (EASO) framework now stresses that “BMI alone is insufficient as a diagnostic criterion, and body fat distribution has a substantial effect on health.”^26^ However, in contrast to cardiometabolic outcomes, the adiposity distribution-cancer relationship has thus far only been evaluated in observational settings where results may reflect residual confounding and/or reverse causation, meaning the causality of these relationships is unknown. In addition, the effects of adiposity distribution on circulating molecular traits—such as sex hormones, inflammatory biomarkers, and metabolic features, including those related to insulin signaling and fatty acid metabolism—in relation to cancer risk are also understudied. Therefore, the mediators of the potential causal relationships between adiposity distribution traits and cancer risk remain unclear. Given the rise in obesity worldwide and its effect on the risks of multiple cancer types, investigating the roles of adiposity distribution and related mediating traits in these relationships thus represents a clinically important step forward in deepening our understanding of which individuals are most at risk for different cancer types, and which interventions are likely to be most effective for cancer prevention.

Currently, there is a lack of large-scale data in which adiposity distribution, cancer-related molecular traits, and cancer status are all measured in the same individuals. In order to evaluate evidence for causal effects of different adiposity distribution traits with the risks of obesity-related cancers, we employed 2-sample Mendelian randomization (MR), a genetic epidemiological approach that uses genetic instruments as proxies to investigate causal relationships between traits.^27^^,^^28^ We used MR to (1) evaluate the effects of 5 adiposity distribution traits on the risks of 12 obesity-related cancers; (2) evaluate the effects of the same adiposity traits on cancer-related molecular traits; (3) evaluate the effects of adiposity distribution-related molecular traits on cancer risk; and (4) estimate the proportion of the effects of adiposity distribution traits on cancer risk that may be mediated by the molecular traits identified.

## Methods

**Figure 1.**
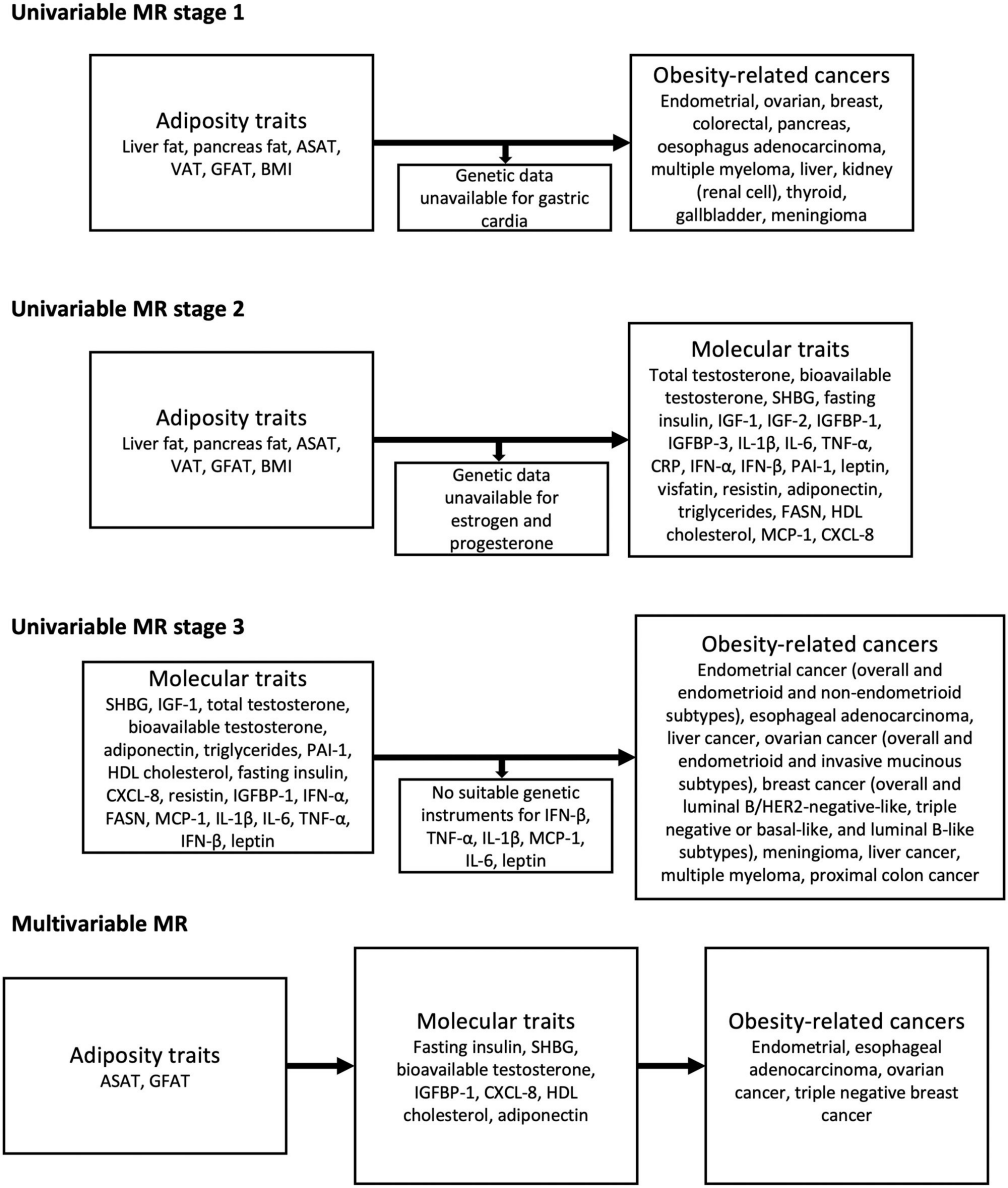
Flowchart detailing analysis plan.

We performed 4 analyses in sequence (Figure 1). First, we performed MR to evaluate the causal effect of 5 adiposity distribution traits and BMI on the risk of all obesity-related cancers described in the 2016 IARC report for which suitable summary genetic data were available (which there were for 12 of the 13 cancers).^4^ Second, we performed an MR analysis to evaluate evidence for the effect of adiposity traits on circulating levels of various molecular traits that are potential mediators of the effects of adiposity measures on cancer risk. Third, we performed an MR analysis of molecular traits for there was evidence for a causal effect of an adiposity distribution trait on, to risks of the adiposity-related cancers. Analyses 2 and 3 represent a two-step MR approach to identify potential mediating traits in these relationships.^29^^,^^30^ Finally, we performed multivariable MR to evaluate and quantify the potential mediating role of these traits in the causal effects of adiposity distribution on the risks of obesity-related cancers.

### Study populations

Table S1 shows the source of genetic data for each trait used in this study. Consent was obtained from all participants, and ethics were approved by the respective institutional review boards. In some instances, there was population overlap between genome-wide association studies (GWAS); in these cases, analyses were repeated using GWAS from independent populations for the adiposity, molecular, and cancer traits. For 6 cancers (Table S2), data from UK Biobank were meta-analyzed with data from FinnGen (discussed below). Given that the adiposity data were also generated using UK Biobank, it is likely that high levels of sample overlap are present for MR analyses using these data. As such, particularly in the presence of weak instruments, estimates may be biased.^31^^,^^32^ Although weak instrument bias is a linear function of the degree of overlap, incorporating a larger sample size (and often thereby increasing the degree of sample overlap) can also increase the strength of the instruments, meaning there is a tradeoff between instrument strength and sample overlap in 2-sample MR analyses.

Summary genetic data for all 5 adiposity distribution traits (unadjusted for BMI in order to avoid collider bias^33^^,^^34^) were obtained from GWAS using UK Biobank.^21^^,^^24^ UK Biobank enrolled more than 500 000 individuals between the ages of 40 and 69 years between 2006 and 2010.^35^ Summary genetic data for the adiposity distribution traits were obtained from GWAS of 38 965 UK Biobank participants who underwent magnetic resonance imaging (MRI) scans, followed by quantification of the adiposity traits using a deep learning model trained on body MRI images.^21^^,^^24^

To the best of our knowledge, individuals with a cancer diagnosis were not excluded from the GWAS of adiposity traits, raising some potential for bias from reverse causation in our analyses. Although the cancer status and history of the participants in the initial UK Biobank imaging cohort is not publicly available, cancer incidence in this group can be estimated using age-matched UK population estimates. The adiposity measures were derived from MRI scans conducted at the imaging assessment visit, at which the median participant age was 65 years.^21^ At this age, cancer incidence in the general UK population is approximately 2%.^36^ However, participants in large cohort studies, including UK Biobank, tend to be healthier than the general population.^37^ Those who attended the imaging visit were healthier still—for example, only 3% were current smokers compared with 11% in the full UK Biobank cohort at baseline—suggesting that the proportion with a cancer diagnosis is likely to be lower than estimates derived from the general population.^38^ Consequently, any bias in our analyses due to reverse causation resulting from cancer cases in these GWAS is likely to be minimal.

Before performing MR analyses, pairwise genetic correlation of the adiposity distribution traits and BMI was calculated using LD score regression^39^^,^^40^ (RG = 0.2 to 0.8, median = 0.5, Figure S1).

We applied a systematic approach to identify suitable summary genetic data for the 13 obesity-related cancers identified in the IARC report,^4^ which is shown in Figure S2. Briefly, where available, summary genetic association data were obtained from cancer GWAS consortia or large-scale meta-analyses. Where such data were not available, a meta-analysis was conducted using summary genetic data from UK Biobank and FinnGen (Table S1). Using this approach, 12 of the 13 cancers identified in the IARC report had summary genetic data available^4^ (Table S1); data were unavailable for gastric cardia (also known as upper stomach) cancer. This cancer often overlaps with esophageal adenocarcinoma (for which data were available), because many cases are junctional involving the cardia. Subtype- or anatomical subsite-specific data were included where available. See Supplementary Methods for more information on the cancer GWAS included in this analysis.

### Meta-analysis of UK Biobank and FinnGen cancer cases

For kidney (renal cell) cancer, thyroid cancer, multiple myeloma, liver cancer, and gallbladder cancer, meta-analyses were performed using METAL v2011-03-25 software. We used the software to combine test statistics and standard errors and controlled for population stratification (using the METAL genomic control feature twice—once for each GWAS individually, and again for the meta-analyzed results, as recommended in the METAL documentation; for more information, see: https://genome.sph.umich.edu/wiki/METAL_Documentation).^41^ We then filtered the resulting meta-analysis results to remove any single-nucleotide polymorphisms (SNPs) with a heterogeneity *P*-value <.05. This may be particularly pertinent for meta-analyses involving FinnGen given that many of the participants in this cohort were hospital-based recruits, which can induce selection bias in case-control outcomes.

### Identification of potentially mediating molecular traits

We evaluated whether the adiposity distribution traits had distinct molecular profiles, and whether these molecular traits were in turn mediating the effects of the adiposity measures on cancer risk. To this end, we identified 26 molecular traits that plausibly mediate the effect of adiposity on cancer risk. This a priori list of traits was identified from the 2018 World Cancer Research Fund (WCRF) Continuous Update Project (CUP) report titled “Body Fatness and Weight Gain and the Risk of Cancer.”^42^ Specifically, we included all molecular traits listed in “Appendix 2: Mechanisms” of this report (see Supplementary Methods for more information). As a systematic approach to identifying suitable summary genetic data to proxy these traits, we used the largest GWAS with publicly available data performed in European populations (>80%). Using such an approach, suitable summary genetic data were available for 24 of these 26 traits (all except estrogen and progesterone). Additional information on the GWAS used for these traits, including sample sizes and ancestry, is available in Table S1.

### Mendelian randomization analyses

We performed MR to evaluate evidence for a causal effect of exposures on outcomes. Mendelian randomization can result in unbiased estimates of causal effects if these assumptions are met: (1) the instrument strongly associates with the exposure; (2) there is no confounding of the instrument-outcome relationship; and (3) the instrument only affects the outcome through the exposure.^43^ Two-sample MR has the additional assumption that the samples used to obtain SNP-exposure and SNP-outcome associations must be representative of the same underlying population.^44^ The STROBE-MR reporting guidelines have been followed throughout this article (Supplementary Note).^45^^,^^46^

To construct genetic instruments for MR analyses, we obtained SNPs strongly (*P-*value < 5 × 10^−8^) associated with each trait. We then performed linkage disequilibrium (LD) clumping to retain only independent SNPs, based on an LD threshold of r^2^ < 0.001 within a 10 000 kb window, with the SNP with the smallest *P*-value retained from each clump. For any trait that is the direct product of a single gene (ie, the proteins IGFBP-1, adiponectin, IFN-β, IL-6, PAI-1, resistin, TNF-α, IL-1β, visfatin, IGF-1, C-reactive protein, MCP-1, leptin, and FASN), we restricted genetic instruments to all remaining *cis*-acting SNPs (those in or within a 1 MB window around the gene coding region) after clumping in order to minimize the risk of horizontal pleiotropy from *trans*-acting variants acting primarily through other genes/proteins. Using this approach, no suitable genetic instruments were available for IFN-β, TNF-α, IL-1β, MCP-1, leptin, or IL-6, meaning these traits were not instrumented as exposures in MR analyses. It should be noted that previous analyses have employed more comprehensive approaches to identify genetic instruments to proxy some of these traits—for instance, by using alternative GWAS in the case of leptin or using SNPs in or near a relevant receptor (and weighting by their effect on C-reactive protein) in the case of IL-6.^47^^,^^48^ Given the large number of traits included in these analyses, we maintained a systematic approach to instrument construction as described above and did not implement previously described approaches to identify instruments for IFN-β, TNF-α, IL-1β, MCP-1, leptin, and IL-6. For sex hormone and related traits (ie, total testosterone, bioavailable testosterone, and sex hormone-binding globulin (SHBG)), genetic instruments were constructed using sex-specific GWAS. The genetic instruments used for all traits are shown in Table S3.

Where there was a single genetic instrument available for a trait, the Wald ratio and delta method were used to calculate effect estimates and approximate standard errors, respectively.^49^ Where there were multiple genetic instruments available, inverse-variance weighted (IVW) multiplicative random-effects models were applied. Where there were more than 10 genetic instruments available for a trait, weighted median estimation and weighted mode estimation were used to evaluate evidence for horizontal pleiotropy (a violation of assumption 3 of MR).^50-53^ As a heuristic, any analyses with a *P*-value below .05 were classed as having evidence for an effect. In addition, we categorized any analysis that passed a Bonferroni correction accounting for multiple testing (*P*-value <.05/29 cancer types (12 overall cancers, plus 17 subtypes); .05/24 molecular traits; .05/21 unique adiposity-cancer pairs; or .05/7 multivariable MR analyses) as having “strong” evidence for an effect. For all analyses where the same exposure (adiposity distribution or molecular trait) was evaluated in relation to an overall cancer type in addition to subtypes (which were available for breast, endometrial, ovarian, and colorectal cancer), we also evaluated pairwise heterogeneity between each subtype and overall cancer type MR estimates using Cochran’s Q statistic, and applied a Bonferroni correction across all heterogeneity analyses.^54^

*F*-statistics were calculated as R^2^ × (N-1)/((1-R^2^) × k, where k is the number of genetic instruments and N is sample size of the exposure GWAS for all SNPs for each individual exposure; any SNP with an *F*-statistic <10 was excluded (and a mean was calculated for each exposure) before conducting MR analyses, to assess potential for weak instrument bias (a violation of assumption 1 of MR).^55^ In addition, Steiger filtering was performed before MR analyses, with any genetic instruments explaining more variance in the outcome than the exposure excluded^56^ (see Supplementary Methods for more information). Post hoc power calculations were performed for all MR analyses evaluating evidence for a causal effect of adiposity measures on cancer risk and for the minimum, maximum, and median sample sizes of molecular traits. For power calculations, a series of odds ratios (IVW ORs) were used to produce power curves demonstrating estimated power in our analyses for a given magnitude of causal effect of the exposure on cancer risk (Figures S3 and S4). It should be noted that these magnitudes of causal effect are subject to the normalization procedure conducted in the GWAS, meaning it is unclear what a biologically relevant magnitude would be.

We employed several sensitivity analyses to evaluate the reliability of our results. We repeated all analyses avoiding sample overlap by using summary-level data for the exposure or outcome that did not contain UK Biobank, as outlined in Supplementary Methods. Additionally, for any MR analyses where at least one of the traits is likely to have sex-specific genetic architecture (eg, sex hormones and related traits), or where one GWAS was limited to a specific sex (eg, breast and endometrial cancer), we repeated analyses with sex-specific GWAS where available for both exposure and outcome, regardless of the strength of evidence in the sex-combined analyses. Table S1 shows further details on the GWAS used for these sensitivity analyses. Note that sex-specific data were used for all sex hormone traits throughout, as described above.

As an additional sensitivity analysis, we aimed to perform multivariable MR to formally evaluate evidence for distinct roles of different adipose depots, particularly those that had similar effect estimates for cancer risk in the univariable MR analyses. However, combining all 5 adipose depots in a single multivariable MR model resulted in conditionally weak instruments (all conditional *F*-statistics <10, median = 5; see Table S4). Therefore, we also calculated conditional *F*-statistics for all pairwise combinations of the traits. We then performed multivariable MR for any pairs of adiposity traits where both had conditional *F*-statistics >10, and where both traits had at least nominal evidence (*P-*value <.05) for an effect on the same cancer in univariable analyses.

### Mediation analysis

For all potential mediating molecular traits, we performed multivariable MR^57-59^ to evaluate evidence for a mediating role and to estimate the proportion of the total effect of the adiposity measure on cancer risk being mediated by each trait. This was calculated using the product of coefficients method,^60^ in which the indirect (mediated) effect is calculated as the product of the effect of the adiposity trait on the potential mediator (from univariable MR) and the direct effect of the candidate mediator on the outcome (from multivariable MR). The total effect was taken from the initial univariable MR analysis, and the proportion mediated was calculated as the ratio of the indirect to the total effect. Standard errors for the indirect effects were calculated using Sobel’s derivation based on the delta method.^61^^,^^62^ If the same adiposity and molecular trait pair was eligible for mediation analysis in both an overall cancer type and one of its subtypes, and there was no evidence of heterogeneity between them (Bonferroni-corrected *P*-value >.05; see above), we excluded the subtype to avoid redundancy and reduced power from smaller case numbers. Conditional *F*-statistics using an assumed covariance of 0 were calculated to evaluate the potential presence of weak instrument bias in these analyses.^58^^,^^63^

### Statistical analysis

All tests of statistical significance are 2-sided. Statistical analyses were performed using R (Vienna, Austria)^64^ version 4.0.2. Univariable MR analyses were performed using “TwoSampleMR”^56^^,^^65^ (version 0.5.6) and MVMR analysis with “MVMR”^66^ (version 0.3). Proxy SNPs were identified using “LDlinkR”^67^ (version 1.2.3), and LD reference panels were compiled using “ieugwasr”^68^ (version 0.1.5). R packages “gwasvcf”^69^ (version 0.1.1), “gwasglue”^70^ (version 0.0.0.9000), “VariantAnnotation”^71^ (version 1.36.0), and “remotes”^72^ (version 2.4.2) were also used for some MR analyses. R packages “ggforestplot”^73^ (version 0.1.0) and “ggplot2”^74^ (version 3.4.2) were used to create the plots used in figures. Some GWAS data were accessed through the OpenGWAS database API^65^^,^^75^ (see Table S1; accessed January 1, 2024). METAL^41^ (version 2011-03-25) was used for the GWAS meta-analyses. All scripts used to carry out this analysis are available alongside a step-by-step walkthrough at: https://github.com/EmmaHazelwood/Adiposity-distribution-cancer-risk-MR.git. Power calculations were performed using https://sb452.shinyapps.io/power/.

## Results

### Evaluating the effect of measures of adiposity on risk of obesity-related cancers

**Figure 2.**
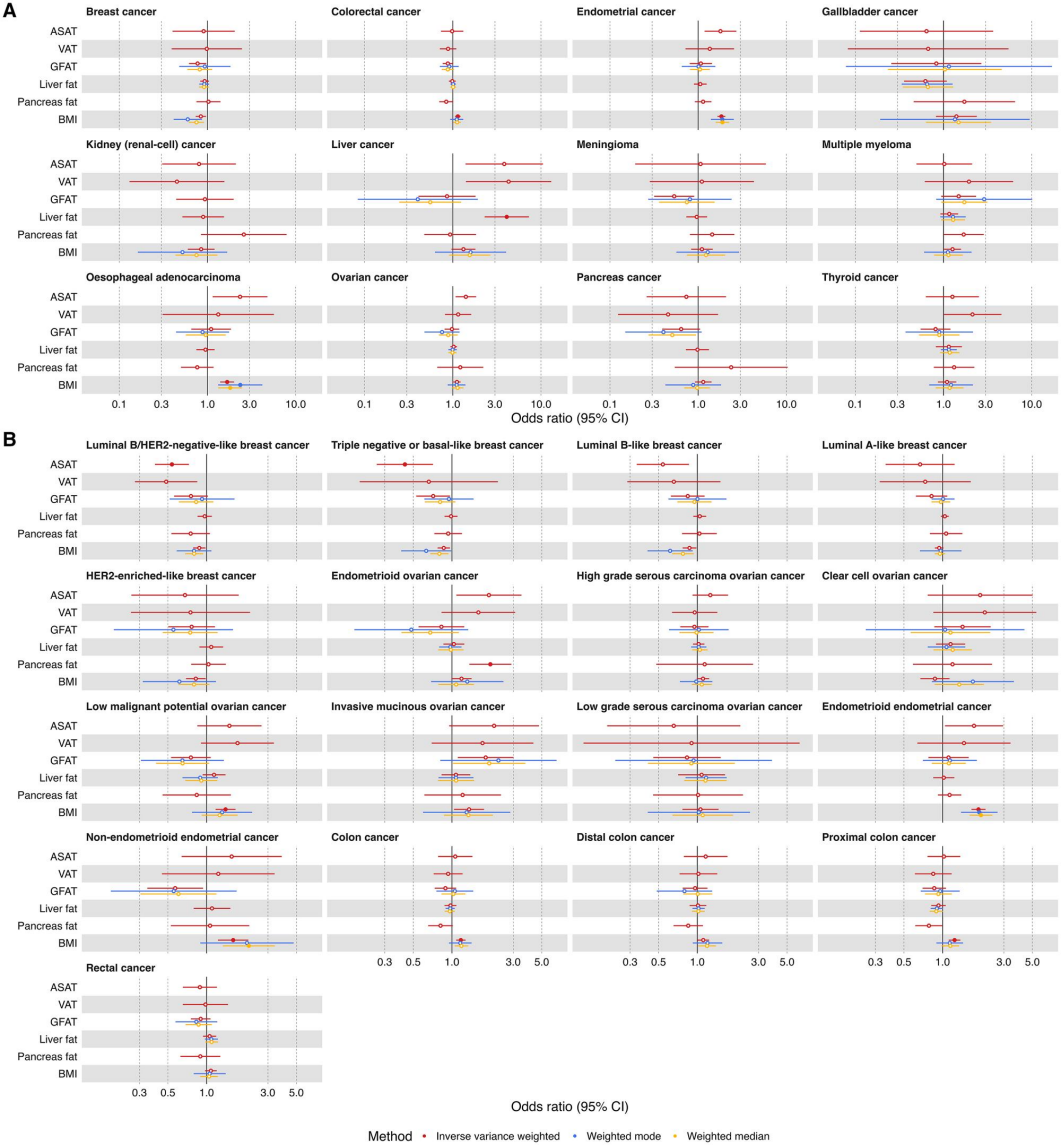
Univariable MR results examining the effect of measures of adiposity on risk of obesity-related cancers, **A)** overall and **B)** by subtypes. Odds ratios shown are given as 1 SD increase in adiposity measure. Open/closed circles indicate the *P*-value did not/did meet the multiple testing-corrected evidence threshold (*P* < .05/12 cancer types), respectively. Abbreviations: ASAT = adipose subcutaneous adipose tissue; BMI = body mass index; GFAT = gluteofemoral adipose tissue; VAT = visceral adipose tissue.

All results are given per standard deviation (SD) increase in the exposure with 95% confidence intervals (CIs); where continuous traits were inverse rank normally transformed before genome-wide analysis we assume the distribution of the traits before transformation was normal and therefore we consider these to be normalized SD units. There was evidence (*P*-value <1.72 × 10^−3^; 0.05/29 cancer types (12 overall cancers plus 17 subtypes)) for an effect of higher genetically predicted liver fat on liver cancer risk (IVW OR = 4.09, 95% CI = 2.29 to 7.28), higher genetically predicted pancreas fat on endometrioid ovarian cancer risk (IVW OR = 1.99, 95% CI = 1.37 to 2.90), and for a protective effect of higher genetically predicted ASAT on luminal B/HER2-negative-like (IVW OR = 0.54, 95% CI = 0.40 to 0.73) and triple-negative or basal-like breast cancer risk (IVW OR = 0.43, 95% CI = 0.26 to 0.71; Figure 2, Table S5). Without multiple testing correction, there was also evidence for a causal effect of higher genetically predicted ASAT, VAT, GFAT, and pancreas fat on risk of several further cancer types (Figure 2, Table S5). The results of the MR analysis evaluating the causal effect of BMI on risk of the 12 cancers and subtypes are provided in Table S6.

Repeating initial MR analyses of VAT on risk of luminal B/HER2-negative-like breast cancer using female-specific data resulted in a different direction of effect than those derived from the primary (ie, sex-combined VAT) analysis. In all other sensitivity analyses, estimates derived from those examining the effect of sample overlap and sex-specific data were in a consistent direction with those derived from the primary analyses, although some 95% CIs crossed the null (Figures S5 and S6, Tables S7 and S8).

Based on these results, we prioritized adiposity trait and cancer pairs to take forward for subsequent analyses as those with a *P*-value <.05. ASAT was paired with luminal B/HER2-negative-like breast cancer, triple-negative or basal-like breast cancer, endometrial cancer, liver cancer, luminal B-like breast cancer, ovarian cancer, esophageal adenocarcinoma, endometrioid ovarian cancer, and endometrioid endometrial cancer. GFAT was paired with invasive mucinous ovarian cancer, meningioma, breast cancer, non-endometrioid endometrial cancer, triple-negative or basal-like breast cancer, and invasive mucinous ovarian cancer. VAT was paired with liver cancer and thyroid cancer. Liver fat was paired with liver cancer. Pancreas fat was paired with endometrioid ovarian cancer, multiple myeloma, and proximal colon cancer.

As a sensitivity analysis, we used multivariable MR to evaluate whether different adipose depots may have independent effects on cancer risk (or whether these effects are shared between depots through the same biological pathways). Specifically, we focused on pairwise combinations of adiposity traits where both traits had strong conditional instrument strength (which we classed as those where both traits have conditional *F*-statistics >10), which were ASAT and liver fat; GFAT and liver fat; GFAT and pancreas fat; and liver fat and pancreas fat (Table S9). Among these, only ASAT and liver fat both showed evidence for an effect on the same cancer—liver cancer—in univariable MR analyses (Figure 2). We therefore conducted a multivariable MR analysis to investigate whether the observed MR effect estimates of ASAT and liver fat with liver cancer risk were driven by shared or distinct biological pathways. In this multivariable MR analysis, the effect estimates for both ASAT and liver fat were attenuated toward the null, suggesting potential overlap in their underlying mechanisms (Figure S8, Table S10).

### Evaluating the effect of measures of adiposity on molecular traits

**Figure 3.**
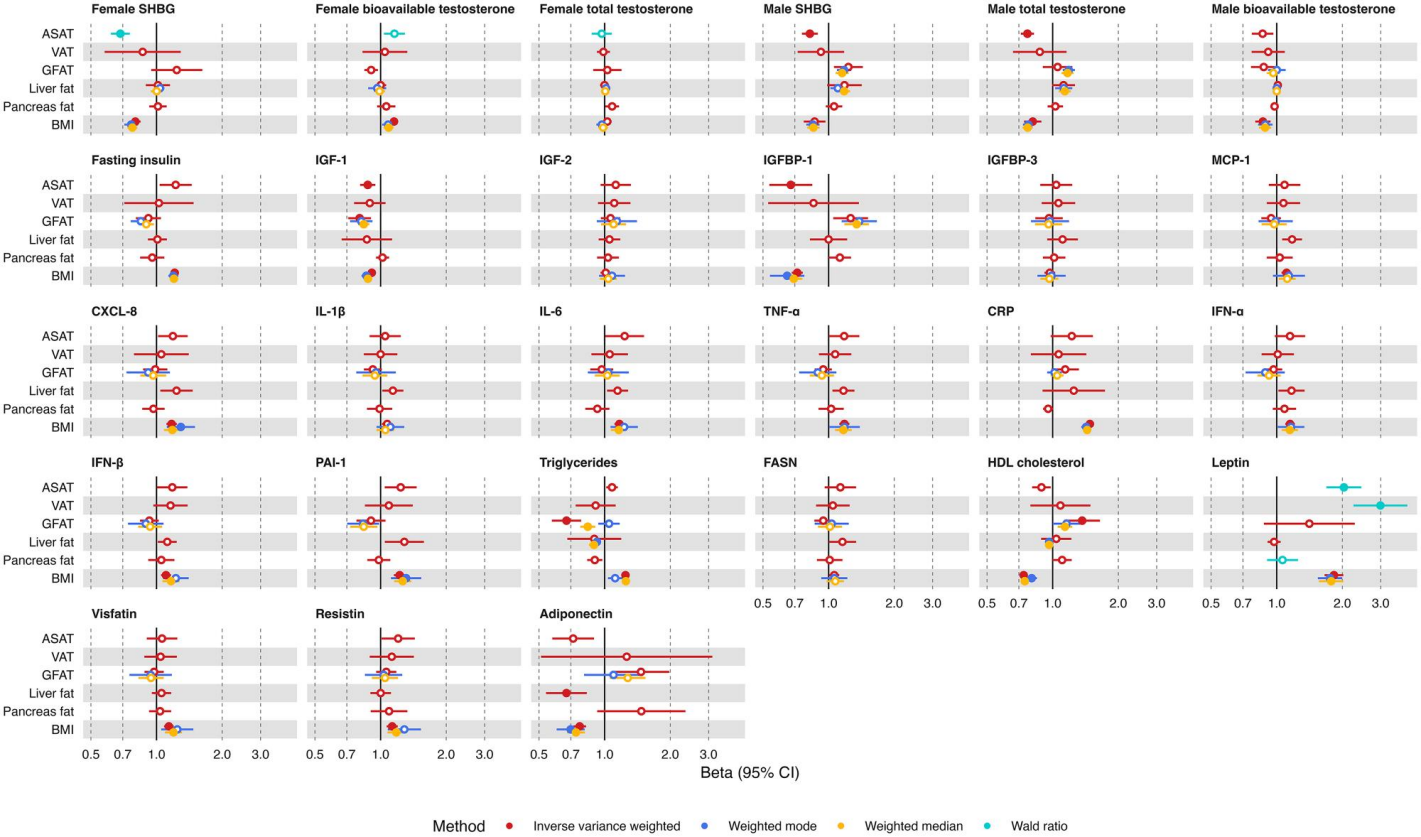
Univariable MR results examining the effect of measures of adiposity on potential molecular mediators of the effect of adiposity on cancer risk. Betas shown are given as 1 SD increase in adiposity measure and inverse-normal transformed nmol/L total testosterone; natural log transformed nmol/L bioavailable testosterone; inverse rank normal transformed SD SHBG; natural log transformed pmol/L fasting insulin; nmol/L IGF-1; SD IGF-2; SD IGFBP-1; SD IGFBP-3; SD MCP-1; SD CXCL-8; SD IL-1B; SD IL-6; SD TNF-a; mg/L CRP; SD IFN-a; SD IFN-B; SD PAI-1; SD triglycerides; SD FASN; SD (0.38 mmol/L) HDL cholesterol; SD leptin; SD visfatin; SD resistin; SD adiponectin. Open/closed circles indicate the *P*-value did not/did meet the multiple testing-corrected evidence threshold (*P* < .05/24 molecular traits), respectively. Abbreviations: ASAT = adipose subcutaneous adipose tissue; CRP = C-reactive protein; CXCL = C-X-C motif chemokine ligand; FASN = fatty acid synthase; GFAT = gluteofemoral adipose tissue; HDL = high-density lipoprotein; IFN = interferon; IGF = insulin-like growth factor; IGFBP = IGF binding protein; IL = interleukin; MCP = monocyte chemotactic protein; PAI = plasminogen activator inhibitor; SHBG = sex hormone-binding globulin; TNF = tumor necrosis factor; VAT = visceral adipose tissue.

All results are given as an SD change in the outcome per SD increase in the exposure with 95% CIs unless units are otherwise stated in Table S1. There was strong evidence (*P*-value <.002; .05/24 molecular traits) for a causal effect of ASAT on leptin (IVW beta = 0.71, 95% CI = 0.53 to 0.89), sex hormone-binding globulin (SHBG; IVW beta = –0.27, 95% CI = –0.38 to –0.15,), IGFBP-1 (IVW beta = –0.40, 95% CI = –0.63 to –0.17), IGF-1 (IVW beta = –0.14, 95% CI = –0.22 to –0.06), total testosterone (IVW beta = –0.07, 95% CI = –0.11 to –0.03); VAT on leptin (IVW beta = 1.10, 95% CI = 0.81 to 1.38); GFAT on triglycerides (IVW beta = –0.40, 95% CI = –0.56 to –0.24), IGF-1 (IVW beta = –0.22, 95% CI = –0.34 to –0.10), HDL cholesterol (IVW beta = 0.31, 95% CI = 0.12 to 0.50); and liver fat on adiponectin (IVW beta = –0.40, 95% CI = –0.62 to –0.19; Figure 3, Table S11). There was evidence (*P* < .05) of potential horizontal pleiotropy for the association between GFAT and triglycerides in that the effect estimate derived using a weighted mode model was in the opposite direction of effect from those derived using the inverse variance weighted and weighted median models. There was also evidence (*P* < .05) for a causal effect of higher genetically predicted ASAT, GFAT, pancreas fat, and liver fat on 18 further molecular traits (Figure 3, Table S11).

In sensitivity analyses examining the effect of sample overlap and sex-specific data, estimates were in a consistent direction with those derived from the primary analysis although some 95% CIs crossed the null (Figures S5 and S7, Tables S7 and S8). The single genetic instrument for female-specific VAT was not available (and no suitable proxies could be identified) for the MR analyses of VAT on female-specific sex hormones, so for these analyses the genetic instrument from the sex-combined VAT GWAS was used.

Based on these results, we prioritized adiposity trait and molecular trait pairs to take forward for subsequent analyses as those with *P* < .05. ASAT was paired with SHBG (female and male), IGFBP-1, IGF-1, total testosterone (male), bioavailable testosterone (female and male), adiponectin, triglycerides, PAI-1, HDL cholesterol, fasting insulin, CXCL-8, and resistin. GFAT was paired with IGF-1, HDL cholesterol, adiponectin, IGFBP-1, SHBG (male), and bioavailable testosterone (female and male). Liver fat was paired with adiponectin, CXCL-8, PAI-1, IFN-α, and FASN. Pancreas fat was paired with triglycerides and total testosterone (female).

### Evaluating the effect of molecular traits on risk of obesity-related cancers

**Figure 4.**
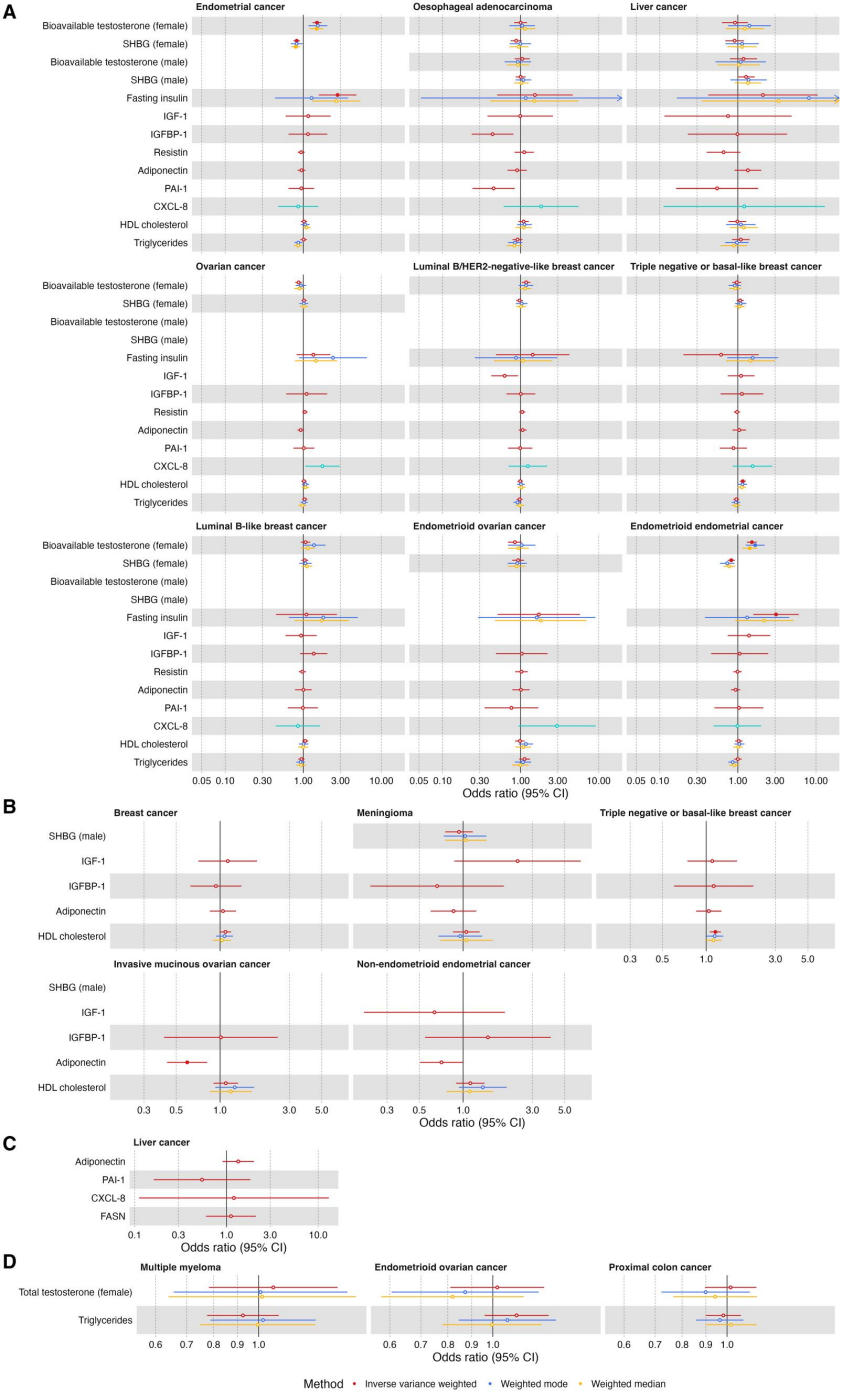
Univariable MR results examining the effect of potential molecular mediators of the effect of adiposity distribution on cancer risk. **A)** Molecular traits and cancers that were found to be affected by ASAT in earlier MR analyses; **B)** molecular traits and cancers that were found to be affected by GFAT in earlier MR analyses; **C)** molecular traits and cancers that were found to be affected by liver fat in earlier MR analyses; **D)** molecular traits and cancers that were found to be affected by pancreas fat in earlier MR analyses. Odds ratios shown are given as increase of one inverse-normal transformed nmol/L total testosterone; inverse rank normal transformed SD SHBG; natural log transformed pmol/L fasting insulin; nmol/L IGF-1; SD IGFBP-1; SD resistin; SD adiponectin; SD PAI-1; SD CXCL-8; SD (0.38 mmol/L) HDL cholesterol; SD triglycerides; natural log transformed nmol/L bioavailable testosterone. Open/closed circles indicate the *P*-value did not/did meet the multiple testing-corrected evidence threshold (*P* < .05/21 unique adiposity-cancer pairs), respectively. Abbreviations: ASAT = adipose subcutaneous adipose tissue; CXCL = C-X-C motif chemokine ligand; GFAT = gluteofemoral adipose tissue; HDL = high-density lipoprotein; IGF = insulin-like growth factor; IGFBP = IGF binding protein; PAI = plasminogen activator inhibitor; SHBG = sex hormone-binding globulin; VAT = visceral adipose tissue.

All cancers and molecular traits with evidence of association with an adiposity measure were investigated for associations with one another. All results are given per SD increase in the exposure with 95% CIs unless units are otherwise stated in Table S1. In these analyses, there was strong evidence (*P* < .002; .05/21 unique adiposity-cancer pairs) for a causal effect of SHBG on endometrial cancer risk (IVW OR = 0.83, 95% CI = 0.76 to 0.90) and endometrioid endometrial cancer risk (IVW OR = 0.82, 95% CI = 0.74 to 0.89), fasting insulin on endometrial cancer risk (IVW OR = 2.75, 95% CI = 1.58 to 4.78) and endometrioid endometrial cancer risk (IVW OR = 3.08, 95% CI = 1.57 to 6.01), and HDL cholesterol on triple-negative or basal-like breast cancer risk (IVW OR = 1.16, 95% CI = 1.06 to 1.26). There was also evidence (*P* < .05) for a causal effect of total testosterone on risk of endometrial cancer; IGF-1 and leptin on risk of luminal B/HER2-negative-like breast cancer; adiponectin on risk of triple-negative or basal-like breast cancer; total testosterone on risk of luminal B-like breast cancer; total testosterone on risk of endometrioid endometrial cancer; and SHBG on risk of non-endometrioid endometrial cancer (Figure 4, Table S12). In general, effect estimates of consistent direction were found in the weighted median and mode models. The genetic instruments for IGF-1 were not available in the GWAS for ovarian cancer, invasive mucinous ovarian cancer, or endometrioid ovarian cancer, and no suitable proxy SNPs were available, meaning the causal effect of IGF-1 on these cancers could not be estimated.

In sex-specific sensitivity analyses, estimates were in a consistent direction with those derived from the primary analysis, although some 95% CIs crossed the null (Table S8, Figure S7). There was not any sample overlap for the MR analyses of molecular traits on cancer risks where evidence for an effect was observed. For the following MR analyses, the direction of the effect of the molecular trait on the cancer risk was not consistent with the direction of the effect of the adiposity distribution trait on the molecular trait and the cancer risk (eg, if the adiposity trait increased levels of the molecular trait and increased risk of a cancer, the molecular trait would need to increase risk of the cancer): bioavailable testosterone (female) on risks of ovarian and luminal B/HER2-negative-like breast cancer, PAI-1 on risk of esophageal adenocarcinoma, IGF-1 on risk of luminal B/HER2-negative-like breast cancer, HDL cholesterol on risk of triple-negative breast cancer, and adiponectin on risk of invasive mucinous ovarian cancer.

Given these results, the following adiposity trait-molecular trait-cancer trios were eligible to be taken forward for mediation analyses: ASAT-fasting insulin-endometrial cancer; ASAT-fasting insulin-endometrioid endometrial cancer; ASAT-SHBG (female)-endometrial cancer; ASAT-SHBG (female)-endometrioid endometrial cancer; ASAT-bioavailable testosterone (female)-endometrial cancer; ASAT-bioavailable testosterone (female)-endometrioid endometrial cancer; ASAT-IGFBP-1-oesophageal adenocarcinoma; ASAT-CXCL-8-ovarian cancer; ASAT-HDL cholesterol-triple-negative breast cancer; and GFAT-adiponectin-non-endometrioid endometrial cancer. In the heterogeneity testing, for all analyses involving the endometrioid subtype of endometrial cancer, there was little evidence that this effect was distinct from the effect of overall endometrial cancer (*P* >3.91 × 10^−4^; .05/128 heterogeneity analyses; Table S13). Therefore, endometrioid endometrial cancer was not included in the mediation analysis.

### Multivariable MR mediation analysis

For all potential biological mechanisms identified (ie, where we found evidence for an effect of an adiposity trait on a molecular trait, and for molecular trait on cancer risk in a direction consistent with the effect of the adiposity trait on cancer risk), we performed multivariable MR to estimate the mediating role of the molecular trait.

**Figure 5.**
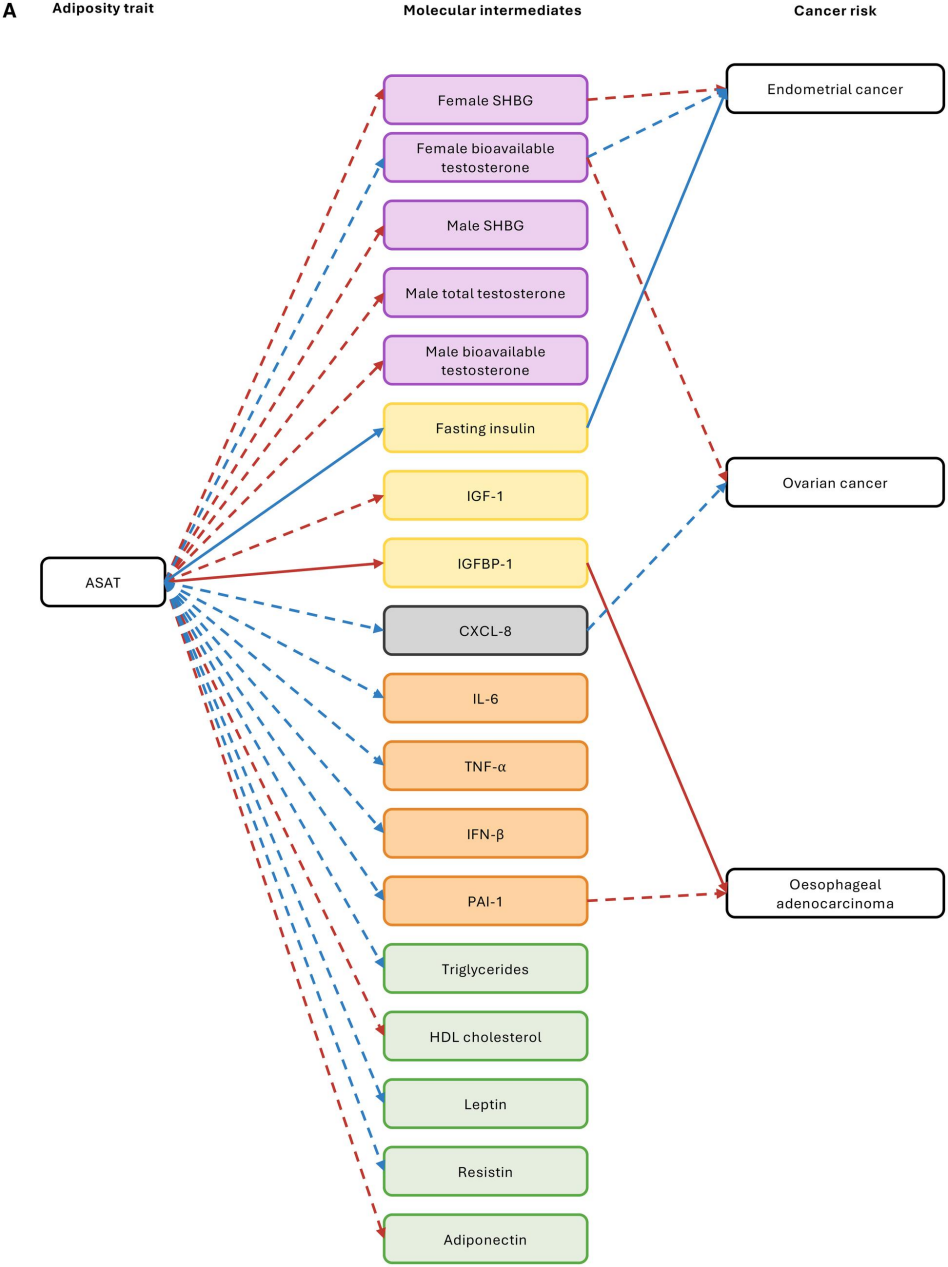

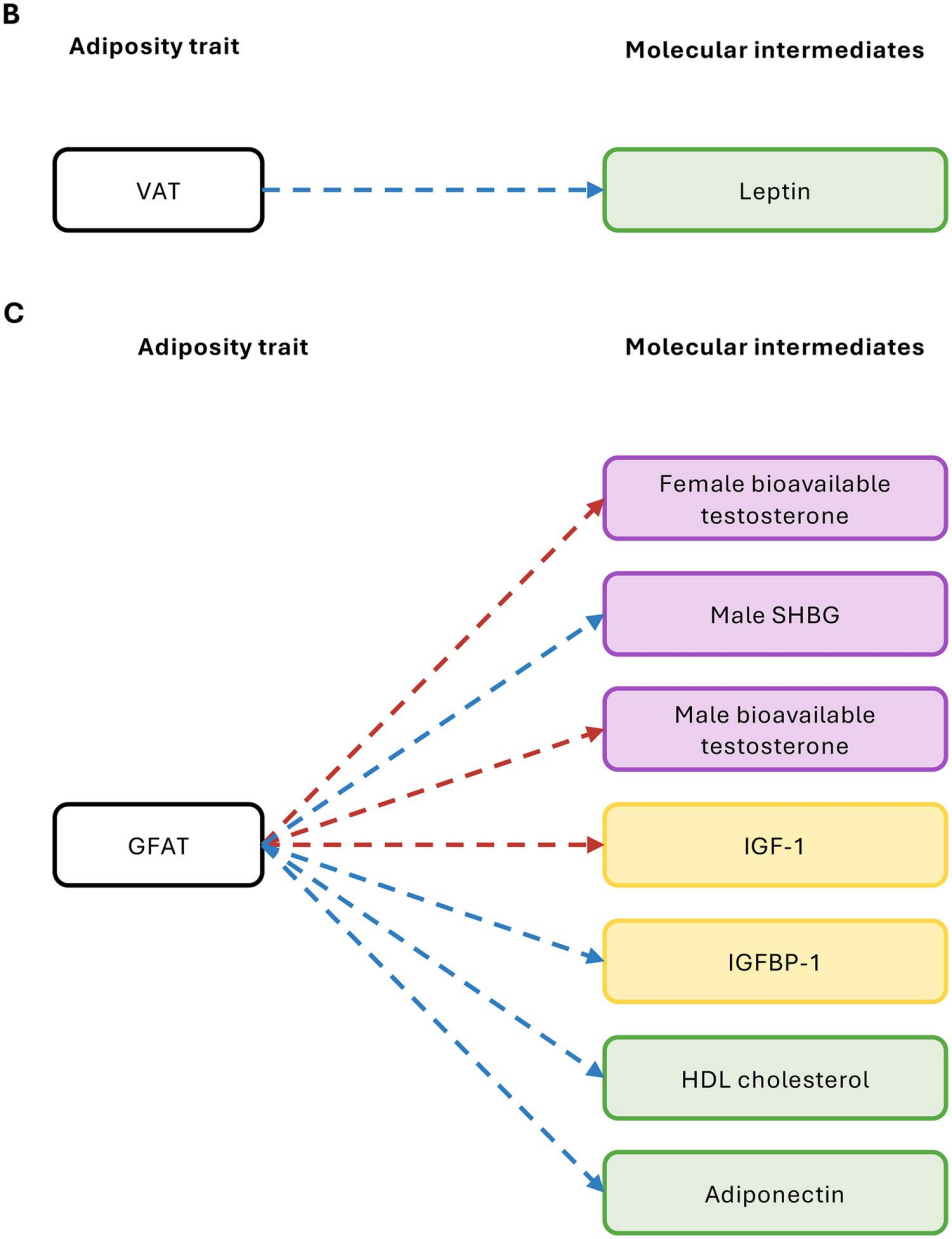

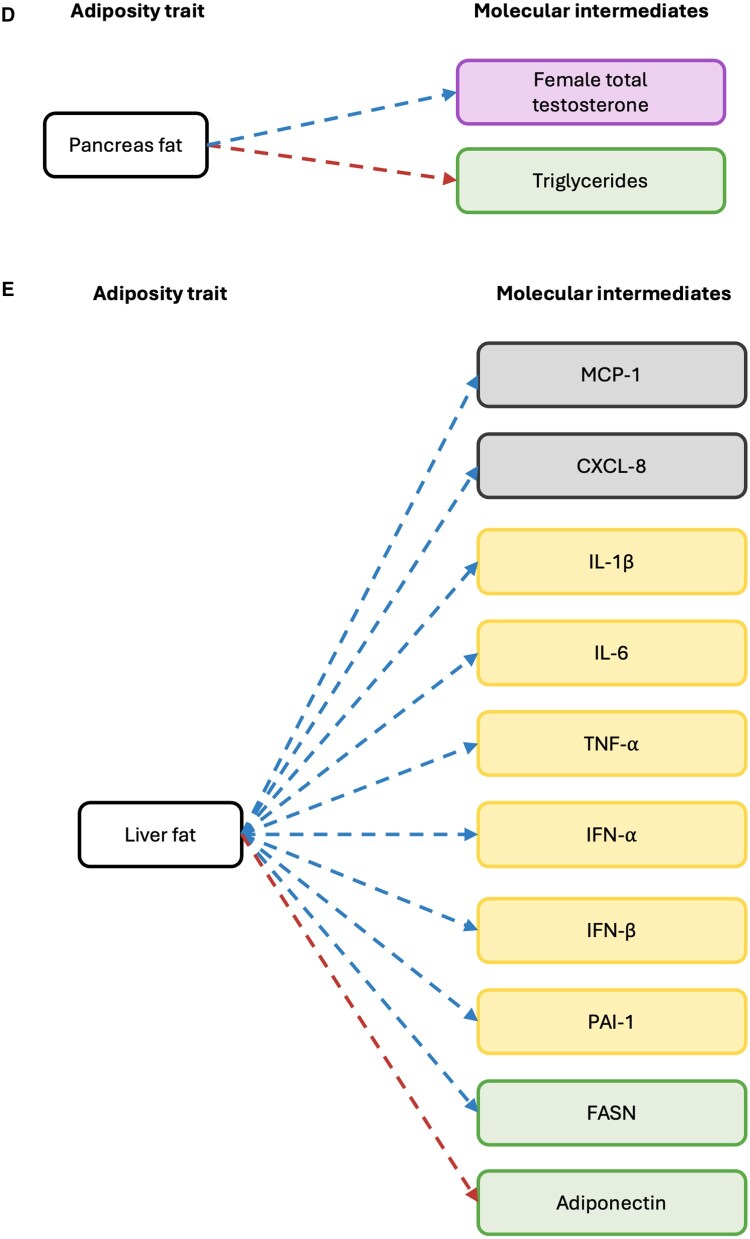
Schematic showing the results from MR analyses of adiposity measures on molecular traits, and molecular traits on cancer risks (overall only, ie, not including cancer subtypes/subsites). Purple molecular traits are sex hormones and related traits; yellow molecular traits are insulin-related traits; orange molecular traits are inflammation-related adipokines; green molecular traits are lipid-related traits; gray traits are chemokine traits. Red arrows represent analyses with evidence for a causal effect that increases molecular trait levels or cancer risk; blue arrows represent analyses with evidence for a causal effect that decreases molecular trait levels or cancer risk. Solid arrows represent analyses for which there was some evidence (*P* < .05) for a mediating effect of the molecular trait in multivariable MR analyses; dotted arrows represent analyses where there was evidence (*P* < .05) in univariable MR analyses only. Note that no molecular trait evaluated was estimated to mediate 100% of the effects of adiposity measures on cancer risks, so other as yet unknown biological pathways presumably exist but are not shown. **A)** MR results from analyses relating to ASAT. **B)** MR results from analyses relating to VAT. **C)** MR results from analyses relating to GFAT. **D)** MR results from analyses relating to pancreas fat. **E)** MR results from analyses relating to liver fat.

**Figure 6.**
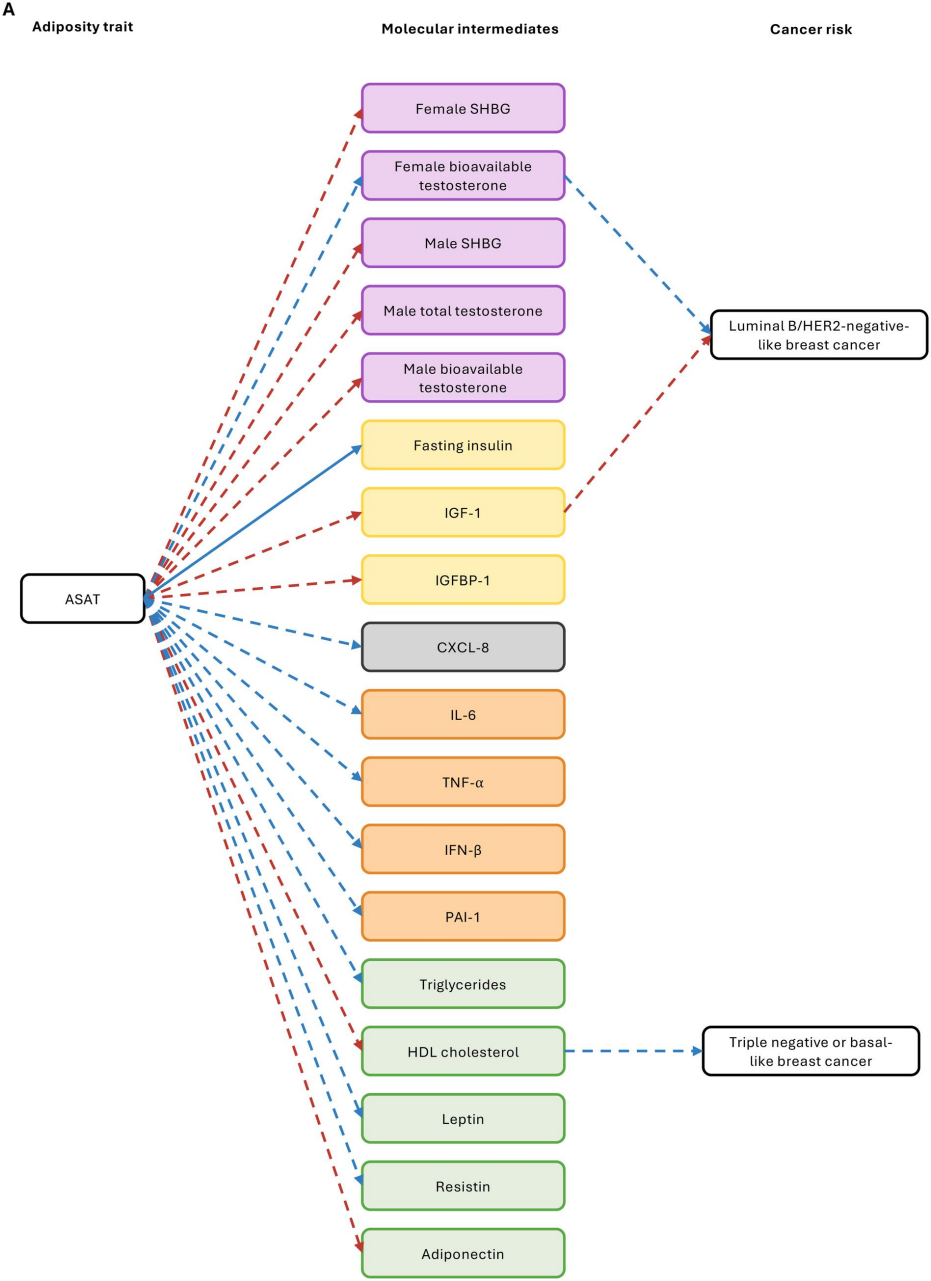

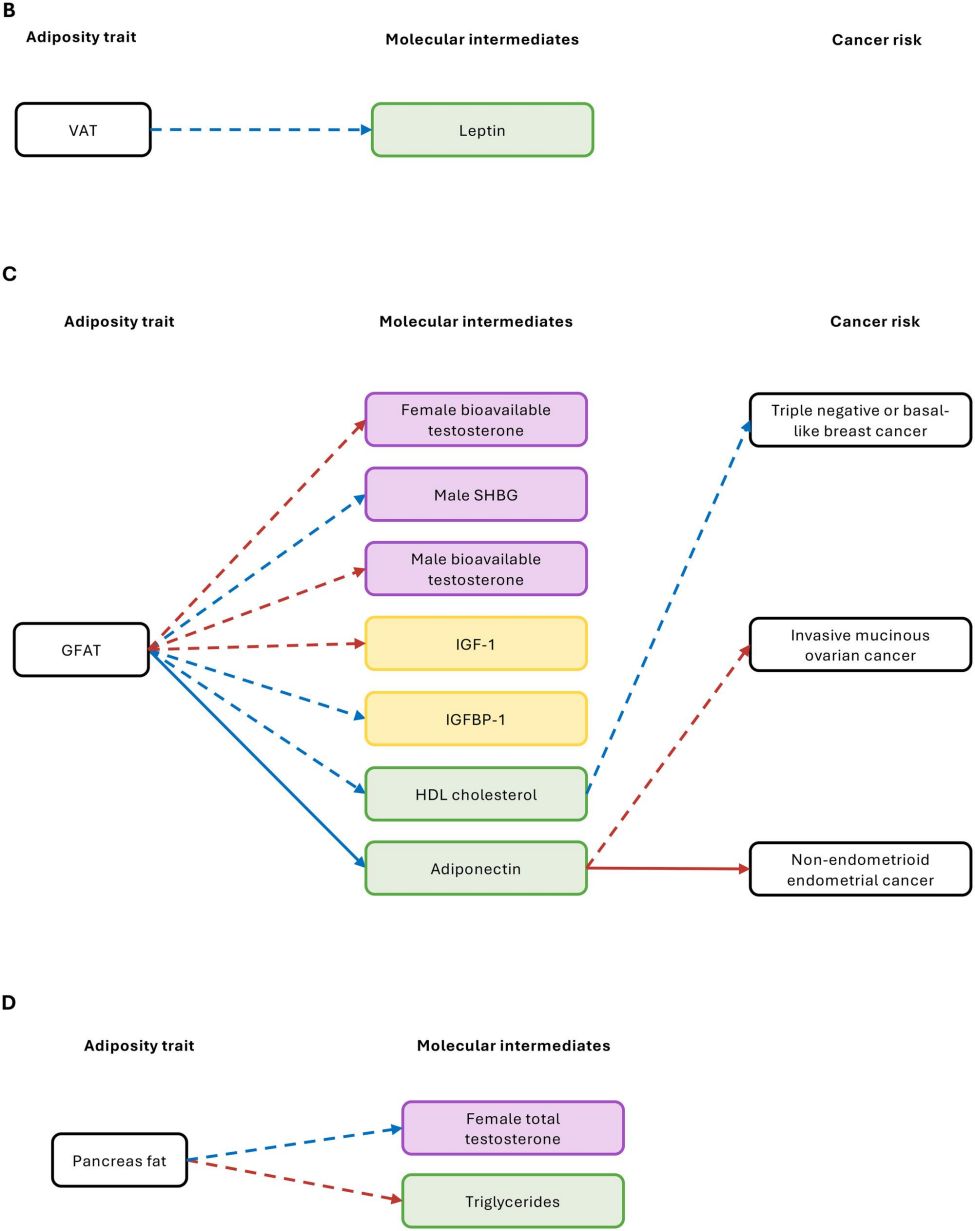
Schematic showing the results from MR analyses of adiposity measures on molecular traits, and molecular traits on subtype/subsite-specific cancer risks. Purple molecular traits are sex hormones and related traits; yellow molecular traits are insulin-related traits; orange molecular traits are inflammation-related adipokines; green molecular traits are lipid-related traits; gray traits are chemokine traits. Red arrows represent analyses with evidence for a causal effect that increases molecular trait levels or cancer risk; blue arrows represent analyses with evidence for a causal effect that decreases molecular trait levels or cancer risk. Solid arrows represent analyses for which there was evidence (*P* < .05) for a mediating effect of the molecular trait in multivariable MR analyses; dotted arrows represent analyses where there was evidence (*P* < .05) in univariable MR analyses only. Note that no molecular trait evaluated was estimated to mediate 100% of the effects of adiposity measures on cancer risks, so other as yet unknown biological pathways presumably exist but are not shown. **A)** MR results from analyses relating to ASAT. **B)** MR results from analyses relating to VAT. **C)** MR results from analyses relating to GFAT. **D)** MR results from analyses relating to pancreas fat.

We found strong evidence (*P* < 7.14 × 10^−3^; 0.05/7 multivariable MR analyses) that approximately 13% of the causal effect of ASAT on increased endometrial cancer risk was mediated by lower SHBG (95% CI = 5% to 20%). However, we note that this analysis may be subject to weak instrument bias (as conditional *F*-statistics for ASAT were <10), which can bias effect estimates toward or away from the null in multivariable MR analyses^66^ (Table 1, Table S14). There was also evidence (*P* < .05) for a mediating role of higher genetically predicted bioavailable testosterone in the effect of ASAT on increased risk of endometrial cancer (percent mediated = 11, 95% CI = 2 to 20%), although this analysis may also suffer from weak instrument bias; lower IGFBP-1 in the effect of ASAT on increased esophageal adenocarcinoma risk (percent mediated = 42, 95% CI = 7% to 77%); higher genetically predicted adiponectin in the effect of GFAT on reduced non-endometrioid endometrial cancer risk (percent mediated = 27, 95% CI = 1% to 52%); and higher genetically predicted fasting insulin in the effect of ASAT on increased risk of endometrial cancer (percent mediated = 35, 95% CI = 1% to 68.1%; Table 1, Table S15). These findings should be interpreted cautiously, given they were not adjusted for multiple testing burden and some may suffer from weak instrument bias. Relationships between adiposity distribution traits, molecular traits, and cancer risks are summarized in Figures 5 and 6. Note that conditional *F*-statistics were also <10 for multivariable MR analyses examining a potential mediating role of CXCL-8 in the effect of ASAT on ovarian cancer risk, and HDL cholesterol in the effect of ASAT on triple-negative or basal-like breast cancer risk, indicating possible underestimation of their mediating roles.

**Table 1.** Results of multivariable MR mediation analyses.

Analysis	Unadjusted OR	Unadjusted 95% confidence interval	Adjusted OR	Adjusted 95% confidence interval	Percent mediated	95% confidence interval for percent mediated	*P* for percent mediated
Exposure 1: ASAT; Exposure 2: SHBG; Outcome: Endometrial cancer	1.79	1.18 to 2.71	1.23	0.99 to 1.52	12.61	5.3 to 19.93	7.23E-04
Exposure 1: ASAT; Exposure 2: Bioavailable testosterone; Outcome: Endometrial cancer	1.79	1.18 to 2.71	1.3	0.98 to 1.73	11.09	2 to 20.17	1.68E-02
Exposure 1: ASAT; Exposure 2: IGFBP-1; Outcome: Oesophageal adenocarcinoma	2.34	1.15 to 4.78	1.64	0.94 to 2.86	42.08	6.74 to 77.42	1.96E-02
Exposure 1: GFAT; Exposure 2: adiponectin; Outcome: Non-endometrioid endometrial cancer	0.57	0.35 to 0.94	0.64	0.43 to 0.94	26.55	0.81 to 52.29	4.32E-02
Exposure 1: ASAT; Exposure 2: fasting insulin; Outcome: Endometrial cancer	1.79	1.18 to 2.71	1.28	0.98 to 1.67	34.53	0.95 to 68.1	4.38E-02
Exposure 1: ASAT; Exposure 2: HDL cholesterol; Outcome: Triple negative or basal-like breast cancer	0.43	0.26 to 0.71	0.82	0.68 to 0.99	2.38	−0.03 to 4.78	5.30e-02
Exposure 1: ASAT; Exposure 2: CXCL-8; Outcome: Ovarian cancer	1.4	1.07 to 1.84	1.25	1.05 to 1.49	28.1	−1.7 to 57.9	6.46e-02

Odds ratios shown are given as 1 SD increase in adiposity measure. Results are ordered by ascending *P*-value; those above the dotted horizontal line passed the multiple testing-corrected *P*-value threshold of 7.14 × 10^−3^.

Abbreviations: ASAT = adipose subcutaneous adipose tissue; CXCL = C-X-C motif chemokine ligand; GFAT = gluteofemoral adipose tissue; HDL = high-density lipoprotein; IGFBP = insulin-like growth factor binding protein; OR = odds ratio; SHBG = sex hormone-binding globulin.

There was potential sample overlap (which can compound weak instrument bias) in multivariable MR analyses evaluating evidence for a causal effect of ASAT adjusted for HDL cholesterol, SHBG, and bioavailable testosterone on cancer risks. Sensitivity analyses examining the impact of sample overlap on multivariable MR analyses involving HDL cholesterol revealed consistent results with those obtained from primary analyses (Tables S15 and S16). Due to the lack of alternative female-specific summary genetic data for SHBG and bioavailable testosterone, sensitivity analyses examining the impact of sample overlap on these traits could not be performed. Given that the assumption of a covariance of 0 is unlikely to be the case where sample overlap exists, we anticipate that we may be underestimating weak instrument bias for multivariable MR analyses including these traits.

## Discussion

We conducted 2-sample and multivariable MR analyses using the largest available GWAS that included ∼40 000 individuals of European ancestry to investigate the role of 5 understudied adiposity traits in the risks of 12 obesity-related cancers and cancer subtypes/subsites. We found evidence for a causal effect of higher genetically predicted liver fat on increased liver cancer risk, higher genetically predicted pancreas fat on increased endometrioid ovarian cancer risk, and higher genetically predicted ASAT on decreased luminal B/HER2-negative-like and triple-negative or basal-like breast cancer risk. We also found evidence for a causal effect of adiposity traits on several more cancer types, with variable directions of effect, and ASAT showing the most consistent evidence for an effect on cancer types, with evidence for a causal risk-increasing effect on 4 of the 12 overall cancer types investigated. We found little evidence for a causal effect of the other adiposity distribution traits investigated, except for liver fat and VAT on liver cancer risk and a protective effect of GFAT on breast and ovarian cancer risks. Subsequent analyses highlighted a potentially mediating role of SHBG in the effect of ASAT on endometrial cancer, although we note the potential for weak instrument bias in this analysis. We also found evidence for a mediating role of bioavailable testosterone in the effect of ASAT on risk of endometrial cancer (although this analysis may also suffer from weak instrument bias), IGFBP-1 in the effect of ASAT on esophageal adenocarcinoma, adiponectin in the effect of GFAT on non-endometrioid endometrial cancer, and fasting insulin and bioavailable testosterone in the effect of ASAT on risk of endometrial cancer.

The importance of adiposity distribution (alongside the importance of total adiposity) in health outcomes is becoming increasingly recognized. This has been driven mostly by previous research on cardiovascular outcomes, which has highlighted seemingly harmful effects of VAT, ASAT, liver fat, and pancreas fat, but protective effects of GFAT.^21^^,^^24^^,^^25^ However, whether the same causal effects of adiposity distribution on cancer risk exist is unclear. Our analysis suggests, for the first time, that this relationship is not as straightforward for cancer outcomes, with causal effects varying for the different adiposity distribution traits by cancer type. For instance, although we found evidence that higher genetically predicted pancreas fat increases the risk of endometrioid ovarian cancer, we found evidence for a protective effect on proximal colon cancer. Similarly, we found evidence for a protective effect of higher genetically predicted ASAT on risks of luminal B/HER2-negative-like and triple-negative or basal-like breast cancer, and yet there was evidence for increased risk of endometrial, liver, esophageal adenocarcinoma, and ovarian cancer. Overall, our results show that the effects of adiposity distribution traits on cancer outcomes appear likely to vary, both by adiposity trait and cancer type.

Evidence for an effect was also seen for liver fat and liver cancer risk. An association between central adiposity (including liver fat) and liver cancer is well established in previous conventional observational analyses.^76^^,^^77^ In the multivariable MR analysis conducted to assess whether the genetic instruments for ASAT and liver fat were capturing shared mechanistic pathways, the effect estimates for both traits were attenuated toward the null compared with the univariable MR analyses. This suggests that the instruments for ASAT and liver fat may reflect overlapping biological mechanisms, potentially related to central adiposity more broadly. This interpretation is supported by the univariable MR analyses, which also identified an effect of VAT on liver cancer risk. Assuming that adiposity traits are approximately normally distributed, around 16% of the population would be expected to have levels more than 1 SD above the population mean. To contextualize the effect sizes in our MR analyses, we used this estimate to calculate the proportion of liver cancer cases that could be attributable to the adiposity distribution traits investigated here. Using an odds ratio of 4—roughly reflecting our findings for ASAT, liver fat, and VAT on liver cancer risk—we estimate that approximately 32% of liver cancer cases could be attributed to individuals with adiposity distribution traits greater than 1 SD above the mean (using the population attributable fraction formula outlined previously^78^). This illustrates the potential population-level relevance of our MR findings, although we note further research is needed to validate the results described here, as well as to investigate how adiposity distribution traits may interact with other risk factors, including alcohol consumption. By contrast, there was little evidence for a causal effect of BMI on liver cancer risk. This underscores the importance of fat distribution, rather than overall body size, in influencing certain outcomes.

Although the cancers investigated in this analysis are recognized as obesity-related cancers,^4^ for some cancers, including colorectal, gallbladder, kidney, and pancreas cancer, we observed little evidence for a causal effect of any of the adiposity distribution traits investigated on cancer risk. This does not necessarily contradict previous findings, because an observational association between higher adiposity and increased cancer risk in the general population does not in itself imply a direct causal relationship across the life course. Observational studies may capture effects of adiposity at specific life stages, or reflect confounding by related lifestyle or socioeconomic factors, which MR is designed to minimize. Furthermore, our analysis focused on adiposity distribution traits—such as ASAT, VAT, GFAT, liver fat, and pancreas fat—rather than total adiposity, and it is possible that the associations with cancer risk reported in previous studies are driven primarily by overall adiposity (as in the case of colorectal cancer, for which we did see evidence for a causal effect, consistent with previous studies,^79^^,^^80^ of higher genetically predicted BMI on risk). Differences in statistical power, cancer subsite or subtype heterogeneity, or the inability to capture dynamic changes in fat depots over time may also contribute to the lack of evidence observed in some cases. Together, these findings highlight the complexity of disentangling the biological pathways through which adiposity affects cancer risk and underscore the importance of considering both total adiposity alongside the distribution of that adiposity in future studies.

As well as evidence for differential effects of adiposity traits across cancer sites, we also found evidence for variable effects on molecular traits. For instance, we found evidence that higher ASAT and GFAT similarly reduced IGF-1 levels, but had opposing effects on IGFBP-1 levels. Likewise, differential effects of the adiposity traits are also seen for the sex hormones, chemokines, fatty acid-related traits, and adipokines investigated. The different adipose depots are known to vary based on the makeup of cells, and thus it may not be surprising that changes to the volume of adiposity at the different sites result in dysregulation of different pathways.^16^ These complex molecular interactions may explain the variable effects on cancer risk seen for each adiposity trait.

We found evidence suggesting a mediating role of SHBG, fasting insulin, bioavailable testosterone, and IGFBP-1 in the effects of ASAT on obesity-related cancers. Our analysis identified a potentially mediating role of SHBG in the effect of ASAT on endometrial cancer risk, and for a mediating role of bioavailable testosterone and fasting insulin in this relationship, although it should be noted that these analyses suffered from weak instruments, and effects may therefore be biased in either direction and so should be interpreted with caution.^66^ These results are similar to those for BMI obtained previously.^19^^,^^47^ Should these results be replicated in future analyses that avoid weak instrument bias, this would fit with the “unopposed estrogen” hypothesis for the development of endometrial cancer, which postulates that endometrial cancer develops as a result of increased estrogen (which is influenced by insulin levels and is a direct product of the aromatization of testosterone) unopposed by SHBG.^47^

We also found evidence suggesting a mediating role of adiponectin in the effect of GFAT on the risk of non-endometrioid endometrial cancer. Adiponectin is an inflammation-related adipokine produced by adipocytes, with a role in insulin sensitivity and fatty acid metabolism.^81-84^ In contrast to BMI, ASAT, and liver fat, we found that higher genetically predicted GFAT increases adiponectin levels, which then may protect from non-endometrioid endometrial cancer development, thus in part explaining a potential protective effect of GFAT on non-endometrioid endometrial cancer risk. Our finding that higher genetically predicted GFAT increases adiponectin levels reflects previous observational analyses^85-87^^,^^87-97^ and reflects the inflammatory profile of the genetic instruments used to proxy GFAT as characterized previously.^21^ Adiponectin is widely recognized as being important for maintaining cardiometabolic health and is also associated with decreased risk of endometrial cancer in observational analyses.^98-103^ Our findings that the protective effect of GFAT on risk of non-endometrioid endometrial cancer may be partly explained by a favorable adiponectin profile fit with these previous analyses. Non-endometrioid endometrial cancers represent a minority of endometrial cancers, and little is known about their etiology. Compared with endometrioid endometrial cancers, which have a very well-established link with obesity, previous evidence for an association between adiposity and non-endometrioid endometrial cancers is less clear. Our results suggest that a plausible reason for this could be that a harmful effect of overall adiposity may be conflated with a protective effect of higher adiposity specifically within GFAT, possibly explaining previous inconclusive results and demonstrating the importance of considering causal effects of distinct adipose deposits.^47^^,^^104-109^

Finally, our analysis suggested that IGFBP-1 may mediate the effect of ASAT on esophageal adenocarcinoma. The leading hypothesis for how obesity may cause esophageal adenocarcinoma is through increased pressure, which can disrupt the gastroesophageal junction and increase risk of gastroesophageal reflux disease, a known risk factor for esophageal adenocarcinoma.^110^^,^^111^ Our finding that ASAT may have a causal effect on esophageal adenocarcinoma fits with this hypothesis. However, we find novel evidence of IGFBP-1 having a potential molecular mediating role in the ASAT and esophageal adenocarcinoma relationship. IGFBP-1 has a long-established inverse association with obesity, which is likely mediated by changes to insulin levels.^112-114^ Consistent with this, the only measure of adiposity that had evidence for an effect on fasting insulin levels in our analysis was also ASAT. Previous observational research has highlighted an association between IGFBP-1 levels or genetic polymorphisms and esophageal cancer risk.^115-117^ The main physiological role of IGFBP-1 is thought to be the binding and regulation of IGFs, including IGF-1.^118^^,^^119^ In our analysis, we found little evidence for a causal effect of IGF-1 directly on esophageal adenocarcinoma risk, suggesting that the biological mechanism linking IGFBP-1 and esophageal adenocarcinoma risk may be independent of its role regulating IGF-1. One possible explanation for this is through IGFBP-1 binding to integrin α5β1, which has a role in modulating cell motility, adhesion, and proliferation, and has been linked previously to several cancers through its role in these processes.^120^^,^^121^ Alternatively, IGFBP-1 may cause esophageal adenocarcinoma through IGF-1 intercellular transport and intracellular activity, which may not be captured by circulating IGF-1 levels.^120-127^ Thus, currently, the relative importance of molecular traits such as IGFBP-1 remains uncertain in comparison with the mechanical effects of increased abdominal obesity in the development of esophageal adenocarcinoma.

One important limitation of the analyses detailed here is that there is some overlap in the genetic instruments used as proxies for the different adiposity traits (Table S3). However, no two adiposity traits share a complete set of SNPs, and where SNPs are shared the variant effect sizes and weightings used in the MR analyses also differ between traits, meaning that even where some SNPs are shared there is still a degree of specificity of genetic instruments. Supporting this, a major finding of our study is that the effect of adiposity on cancer risk seems to vary substantially depending on anatomical location of adipose depots, suggesting that genetic instruments for adiposity traits are capturing distinct biological signals. Future research should aim to further decipher the different signals being captured.

In terms of further limitations, our analyses were almost exclusively restricted to individuals of European ancestry, which limits the generalizability of our findings to other populations. Additionally, our analyses were limited by the availability of summary genetic data. For many cancer sites investigated in our analyses, there were no large-scale GWAS available, and thus these analyses likely lack power due to few cases being available in UK Biobank and FinnGen. Due to a lack of GWAS data in large, non-overlapping samples for traits, substantial sample overlap exists for several MR analyses described here. Although recent evidence suggests that this is unlikely to be a major issue in the absence of weak instrument bias, several of our multivariable MR analyses also suffered from conditionally weak instruments, potentially compounding this bias.^31^ Similarly, although sex-specific sensitivity analyses were performed where data were available, these data were not available for all traits where there may be heterogeneity of instrument effects by sex. Where data were available, in several of our sex-specific sensitivity analyses we found evidence for a causal effect where little evidence was seen in the primary analysis (for instance, we found evidence for an effect of female-specific but not sex-combined fasting insulin on risk of luminal B/HER2-negative-like breast cancer risk). This suggests that our use of genetic data derived from sex-combined GWAS for traits (other than sex hormones and SHBG) may be obscuring important biological mechanisms. Alcohol consumption was not included as a covariate in the GWAS of adiposity traits, including liver fat, from which the genetic instruments were derived. Future studies should explore potential interactions between genetically predicted adiposity and alcohol consumption in relation to cancer risk, particularly liver cancer. Furthermore, we were unable to include two potentially mediating molecular traits, estrogen and progesterone, in our analysis due to a lack of suitable genetic data. Several further traits in our analysis lacked suitable genetic instruments (as identified through our systematic approach); therefore, evidence for their causal effect on cancer outcomes could not be evaluated, although they could be included as outcomes.

Another limitation of our MR analyses is that for many, we did not have enough genetic instruments to perform pleiotropy-robust models of MR, meaning the potential for violation of one of the key assumptions of MR could not be assessed. Moreover, the MR models used in our analyses assume linear relationships between exposures and outcomes, which is unlikely to be the case for all relationships investigated. MVMR additionally assumes that there is no exposure-mediator interaction, which cannot currently be assessed using summary-level data.^60^ Furthermore, the summary genetic data for the adiposity traits were obtained using a deep learning model, an approach that has been shown to possibly introduce bias in SNP-exposure estimates.^128^ Finally, we investigated circulating molecular traits as potential mediators of the effects of measures of adiposity on cancer risk. However, this does not allow for the investigation of local effects of adipose depots on local intermediates, nor for effects on activity and transport as opposed to total amount of molecular traits. In addition, further molecular or nonmolecular traits (eg, mechanical effects in the case of esophageal adenocarcinoma) may have a role in mediating the effects of measures of adiposity on cancer risk but were not included in this analysis.

A benefit of using summary-level data is that it overcomes the need for all traits (ie, adiposity distribution, molecular traits, and cancer risk) to be measured in the same individuals. Although under certain assumptions MR can evaluate evidence for a causal effect between traits, the relationship between adiposity distribution and cancer risk is complex, with some level of pleiotropy between the different adiposity traits, as well as between adiposity distribution and molecular traits. Therefore, using MR alone, it can be difficult to ascertain directions of effect and true estimations of magnitudes of effect. Thus, future research warrants investigation of the putative causal relationships suggested here in high-quality prospective studies.

## Conclusion

This analysis provides valuable insight into the potential causal effects of different anatomical locations of adipose tissue on obesity-related cancer risk, and highlights some of the potential mechanisms that may explain these effects. We show that the relationship between adiposity distribution and cancer risk is complex, with variable effects depending on the location of the adipose deposit and cancer type. Although future research is required to fully understand these relationships, this has important clinical implications for the management of obesity in cancer prevention. Our results suggest that evaluating changes in adipose tissue distribution may have a role in future obesity treatment and cancer prevention interventions.

## Supplementary Material

djaf201_Supplementary_Data

## Data Availability

The sources for all GWAS data used in this study are listed in Table S1. The data underlying this article are available in the article and in its online supplementary material. All GWAS data generated are available in the GWAS catalog (https://www.ebi.ac.uk/gwas/), under study IDs GCST90570370-GCST90570374.
