# Supplementary material for: Adiposity distribution and risks of 12 obesity-related cancers: a Mendelian randomization analysis
Source: J Natl Cancer Inst. 2025 Sep 24;117(12):2621–42. doi: 10.1093/jnci/djaf201 (PMC12682385; doi:10.1093/jnci/djaf201)
Supplement: djaf201_Supplementary_Data [file djaf201_supplementary_data.zip › Supplementary Materials.docx]

**Supplementary Materials**

**Table S1.** Details of GWAS used in MR analyses. GWAS = genome-wide association study, MR = Mendelian randomization, BMI = body mass index, SHBG = sex hormone-binding globulin, HDL = high-density lipoprotein.

**Table S2.** Details of cancer cases and controls from UK Biobank and FinnGen which were meta-analysed.

**Table S3.** Genetic instruments used for each trait in MR analyses.

**Table S4.** Conditional F-statistics in multivariable MR combining all five adiposity distribution traits.

**Table S5.** Results of MR analyses examining the effect of measures of adiposity on risk of obesity-related cancers.

**Table S6** Results of MR analysis evaluating evidence for a causal effect of BMI on risks of obesity-related cancers.

**Table S7.** Results of sensitivity analyses examining the effect of sample overlap in MR analyses of adiposity traits and cancer-related molecular traits.

**Table S8.** Results of sex-specific MR analyses of adiposity trait, cancer-related molecular traits, and cancer risk.

**Table S9.** Conditional F-statistics in multivariable MR combining all pairwise adiposity distribution traits.

**Table S10.** Results of multivariable MR mediation analyses examining effects of ASAT and liver fat on liver cancer risk.

**Table S11.** Results of MR analyses examining the effect of measures of adiposity on potential molecular mediators of the effect of adiposity on cancer risk.

**Table S12.** Results of MR analyses examining the effect of potential molecular mediators of the effect of adiposity distribution on cancer risk

**Table S13.** Results of overall and subtype cancer heterogeneity testing.

**Table S14.** Conditional F-statistics in multivariable MR analyses evaluating a mediating role of molecular traits in the adiposity distribution-cancer risk relationship.

**Table S15.** Results of multivariable MR mediation analyses examining potential bias from sample overlap.

**Table S16.** Conditional F-statistics in multivariable MR analyses examining potential bias from sample overlap.

**Supplementary note.** MR-STROBE checklist.

**Supplementary methods.**

**Figure S1.** Genetic correlation between adiposity distribution traits. ASAT = adipose subcutaneous adipose tissue; VAT = visceral adipose tissue; GFAT = gluteofemoral adipose tissue; BMI = body mass index.

**Figure S2.** Overview of summary genetic data selection process for obesity-related cancers.

**Figure S3.** Results of power calculations for MR analyses estimating the causal effect of adiposity distribution traits on risk of obesity-related cancers. ASAT = adipose subcutaneous adipose tissue; VAT = visceral adipose tissue; GFAT = gluteofemoral adipose tissue.

**Figure S4.** Results of power calculations for MR analyses estimating the causal effect of adiposity distribution traits on levels of molecular traits, using the maximum (441,016), median (61,983), and minimum (35,559) sample sizes for the traits. ASAT = adipose subcutaneous adipose tissue; VAT = visceral adipose tissue; GFAT = gluteofemoral adipose tissue.

**Figure S5.** Results of repeating univariable MR analyses without sample overlap. Odds ratios/betas shown are given as one SD increase in adiposity measure. Open/closed circles indicate the *P*-value did not/did meet the evidence threshold (*P*-value < 0.05) respectively. ASAT = adipose subcutaneous adipose tissue; VAT = visceral adipose tissue; GFAT = gluteofemoral adipose tissue; IGF = insulin-like growth factor; HDL = high-density lipoprotein.

**Figure S6.** Results of sex-specific univariable MR analyses of measures of adiposity on cancer risk; (A) overall and (B) by subtype. Odds ratios shown are given as one SD increase in adiposity measure. Open/closed circles indicate the *P*-value did not/did meet the evidence threshold (*P*-value < 0.05) respectively. ASAT = adipose subcutaneous adipose tissue; VAT = visceral adipose tissue; GFAT = gluteofemoral adipose tissue; BMI = body mass index.

**Figure S7.** Results of sex-specific univariable MR analyses of molecular traits on cancer risk. Open/closed circles indicate the *P*-value did not/did meet the evidence threshold (*P*-value < 0.05) respectively. ASAT = adipose subcutaneous adipose tissue; VAT = visceral adipose tissue; GFAT = gluteofemoral adipose tissue; SHBG = sex hormone-binding globulin; IGF = insulin-like growth factor; HDL = high-density lipoprotein.

**Figure S8.** Univariable and multivariable estimates of ASAT and liver fat on risk of liver cancer. Open/closed circles indicate the *P*-value did not/did meet the evidence threshold (*P*-value < 0.05) respectively. ASAT = adipose subcutaneous adipose tissue.
