# Supplementary figures and images for: Adiposity distribution and risks of 12 obesity-related cancers: a Mendelian randomization analysis
Source: J Natl Cancer Inst. 2025 Sep 24;117(12):2621–42. doi: 10.1093/jnci/djaf201 (PMC12682385; doi:10.1093/jnci/djaf201)

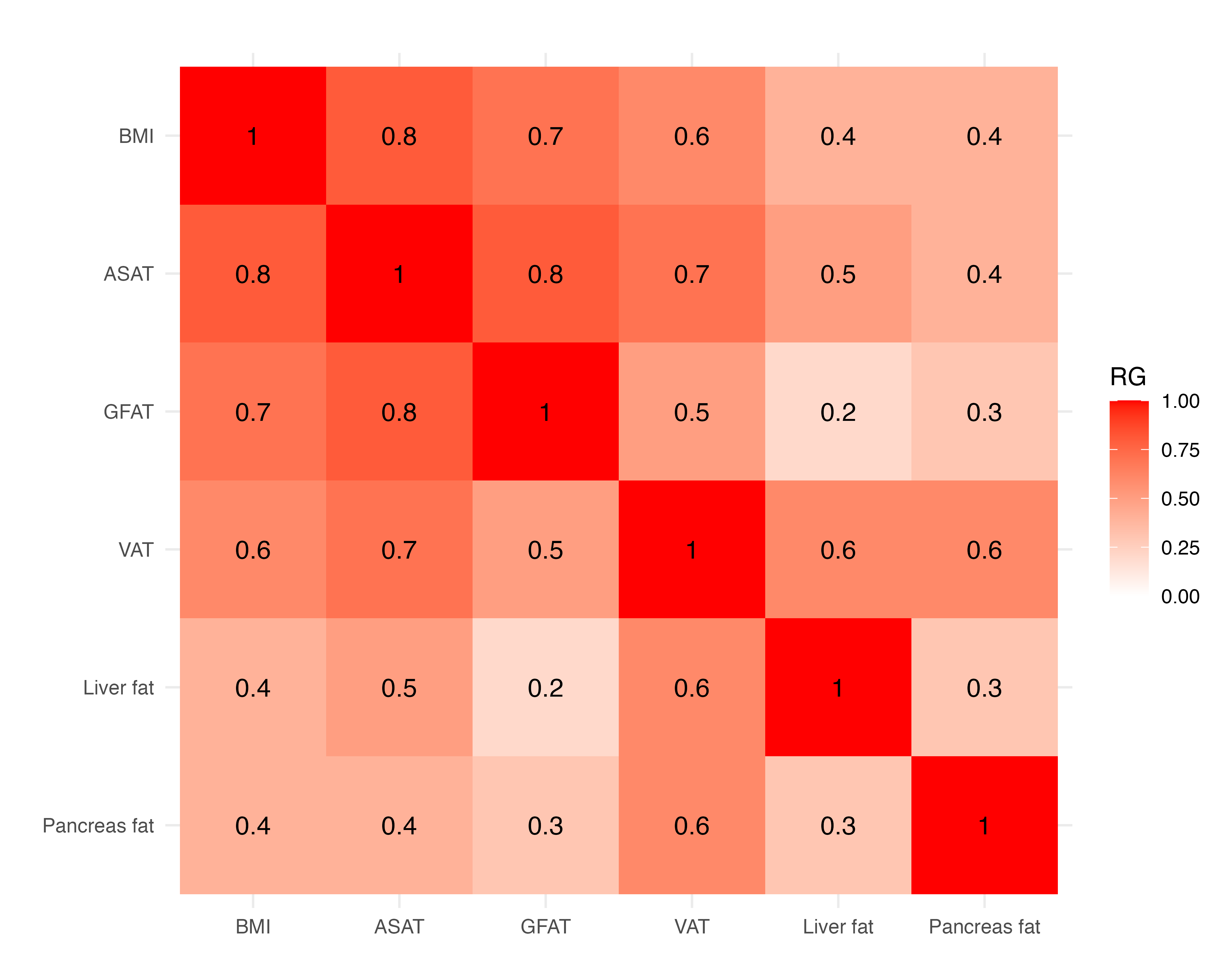

Supplement: djaf201_Supplementary_Data [file djaf201_supplementary_data.zip › Figure S1.png]

**For all obesity-related cancers from 2016 IARC report:**

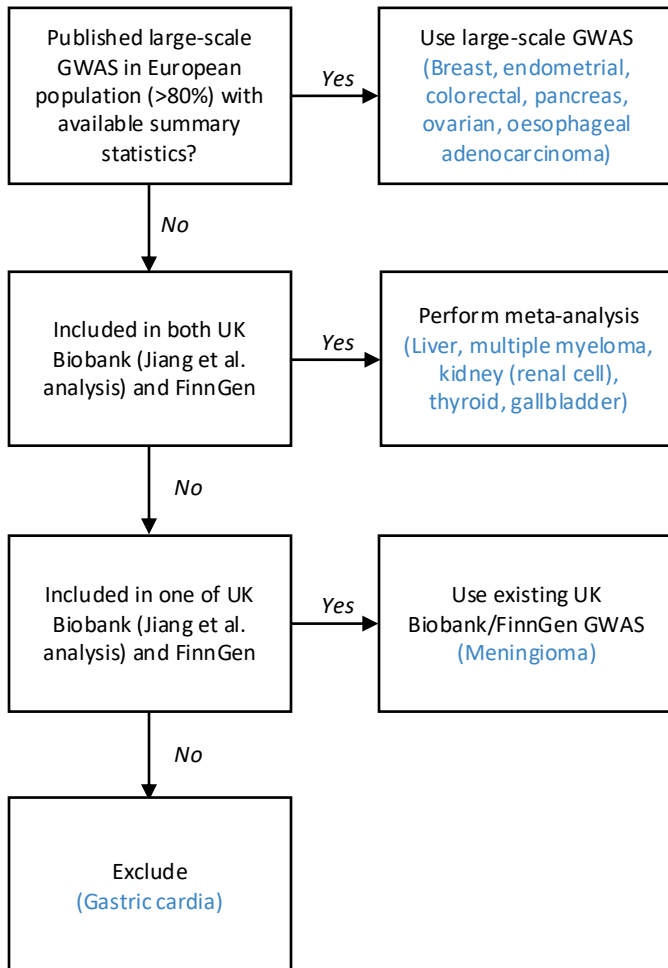

Supplement: djaf201_Supplementary_Data [file djaf201_supplementary_data.zip › Figure S2.pdf]
